# Visiting Day

**Published:** 1887-09-03

**Authors:** R. G. Emery


### VISITING DAY.

By R. G. Emery.

Who, that has passed the great doors of a hospital on a Sunday, or mayhap on a week-day afternoon, has not had his attention drawn to a small knot of people, all in their best clothes, gathered round the portals, and all evidently intent upon some important errand? This spectacle, with the addition of a somewhat unusual number of flower-girls, denotes that it is one of the visiting days at the hospital, of which at every institution there are three or four in each week, and Sunday is invariably one. The Sabbath is, of course, the favourite visiting day: for few people—except it be doctors, journalists, and omnibus men—are so hardworked but that they can devote Sunday afternoon to visiting a friend or a relative who happens to be an inmate of one of the hospitals. Therefore it is that for about a couple of hours on the afternoon of the Day of Rest, most of the wards of the London hospitals are nearly as crowded as a fashionable lady's "at home"; for full advantage is taken of the permission given to each patient to see two visitors. Even on the other visiting days the attendance of patients' friends is by no means small; and anybody who is fond of the study of human nature will find a rich field of observation open to him, if he cares to spend an afternoon, now and again, at these patients' "receptions."

It was in the pursuit of this study that I found myself the other day on my way to St. George's Hospital. It was a May day of the new and improved type—that is to say, wet and dismal—so I sought the shelter of a 'bus, which was filled with people, mostly women, evidently bound to the same institution. "A fellow-feeling," we all know, "makes us wondrous kind," and most of these female travellers were in the full exchange of confidences. One was going to see her little son, and told, in homely, pathetic language, of his patience under his sufferings. Another, whose husband was an inmate of the hospital, was discoursing to her neighbour of her "man's" case. So far as I could learn, he had had to undergo an amputation of the arm for chronic rheumatism, or gout, and the good soul was repeating the story he had told her of his sensations in the shortened limb: for he was rapidly approaching convalescence, and had entertained her with this history when she went to see him on the previous Sunday. The wife seemed to be so overjoyed at the prospect of his speedy discharge, that she made light of the operation, and retailed her narrative with an animation that was amusing to witness. Another conversation on a somewhat similar topic was proceeding in the far corner of the 'bus, but the noise prevented my hearing anything of it.

Arrived at the hospital doors, the little crowd which I have previously mentioned as being usually observable on such a day, was conspicuious by its absence. The pitiless rain drove everybody under cover, and even the flower-girls were glad to seek the shelter of the friendly porch, from which the kind-hearted janitors did not attempt to remove them. I did not observe that they enjoyed a very brisk trade,—and, indeed, to purchase threepenny or twopenny nosegays would have been worse than carrying coals to Newcastle; for St. George's is in the happy position of being the recipient of many floral offerings from the wealthy of the neighbourhood, and the treasures of a score of greenhouses were to be seen displayed on the ward-tables. Many of the visitors had brought simple bouquets, however, and I do not know that the patients prize them less than the more expensive and intrinsically beautiful gifts. Sentiment counts for a good deal, especially if one is sick, and the humble bunch of primroses or narcissi brought by the hand of father or mother, wife or husband, perhaps gives more pleasure than the decorative offerings of kind-hearted strangers. Inside the great hall is a collection of some thirty or forty people waiting to go upstairs. A very brief inquiry on the part of the porters is sufficient to obtain the required permission; and those whose friends have been in-patients for some little time are so well known to the attendants that no interrogation is necessary, and, with a smile and a nod of welcome, they pass through the vestibule and up the stone staircase.

At St. George's the visiting days are four per week, but even this restriction is not adhered to with adamantine rigour, and consequently, except on Sunday, there is very little crowding in the wards. But, seeing that the institution is in the happy position of being able to use all its beds, and that there are seldom fewer than 350 patients within its walls, there is always a very fair sprinkling of visitors; and as one walked from ward to ward one heard the sound of pleasant conversation, with here and there a low laugh of pleasure, and very little of the painful quietude usually associated with the apartments of a great hospital. I saw both my friends of the omnibus, the man with his amputated arm looking extraordinarily happy, and very little the worse for his operation; indeed, I overheard him telling his wife that the doctor had promised that in all probability he would be "out" by the end of the week. In very different case was the other woman, who had come to see her sick child. The poor little fellow was evidently very ill, and it will require all the skill of the medical staff, I fear, to ensure his ever returning to his home. What wee little things some of the patients were, too! Sometimes they are so young that the mothers have to be taken in with them, and their services are utilised in the work of the wards. One baby-girl had plasters across her face in the form of a big star, some operation on the structural bones of the nose having been performed. Her father and mother had come to visit her, and the three-year-old mite seemed as pleased as any of her elders that she was able to see again faces she knew and loved.

In the accident ward, which contained the usual complement of street casualties and mishaps in connection with new buildings—St. George's being far removed from those scenes of manufacture which eastern and southern London present in such variety, seldom has other accidents than these brought to its doors—I was struck with one little fellow, about six or seven years of age, who, it was easy to see, had been run over in one of the busy thoroughfares outside, and had got his leg broken. His father and mother had come to see him. Absurdly young they both looked, to be the parents of a seven years old boy, and I watched them as they sat, one on either side of the bed, without speaking either to each other or to the patient, for quite four or five minutes. The lad himself, if the truth must be told, looked by far the least miserable of the three; and this peculiarity has often struck me before in my peregrinations through one hospital and another. The infantile and youthful in-patients, as soon as they get the least bit convalescent, are undoubtedly, in ninety-nine cases out of a hundred, better off there than at home. A sick child, even though it be ugly—that is, if an ugly child be possible: a point I have often heard kind-hearted matrons hotly dispute—is always interesting, and very often most lovable. Accordingly, all the good things of the hospital life fall abundantly to his or her share, and the happiness depicted on the faces of the child-inmates is not at all to be wondered at. But this is a digression. In the very next room I came across one who had pretty nearly reached the end of the seventh act in life's drama—an old woman visited by her daughter, who, as she talked to the patient, held her baby to her breast. I should have liked to inquire into their history had I dared. The old woman looked as if life for her had been one long battle; nor had the combat been gentle, apparently, with the younger one, whilst the baby was but meanly clad. Yet care was apparently forgotten just then, and the two women conversed in a low, eager tone, which proclaimed to all and sundry that they were determined to make the most of the short visiting hour.

If, however, visiting day is a pleasure to those patients who have visitors, it does not appear to bring much joy to the friendless ones, some of whom wore on their faces an expression very like envy towards their more fortunate fellows. Human nature is the same inside as outside the walls of a hospital, and the visitorless patient, especially if she be a woman, no doubt feels the loneliness more when friends are seated near all the neighbouring beds, whilst her own bedside is empty.

One more incident, and I have done. Having occasion to call at the secretary's offices, it happened that I entered the door at the same time as a patient who was just about to be discharged. He was of the working-class—so much was evident from his manner and speech—but his period of confinement had been long, and all the horny-handedness had disappeared, his palm and fingers being as plump and soft as a lady's. He had come to get an order for some appliance of mechanical surgery, and between him and the clerical official, who was seated at a desk, the following conversation took place:

"So you're going out. Have you been comfortable since you've been here?" "Yes" (this with very great emphasis). "Are you better?" "Oh, yes! I shall soon be as strong as ever I was." "And you've no complaint to make of anything or anybody?" The man looked for a moment as if he thought his interrogator was poking fun at him; but observing that the gravity of the gentleman's face remained unshaken, he replied, "Complaint, sir! Nothing whatever." Then he took his ticket and went out.

When I passed into the street the rain had ceased and the sun was shining brightly. The devotees of *la haute politique* were hurrying down to the House, and the Row was beginning to show signs of animation.

